# On the Anatomy and Diseases of the Neck of the Bladder, and of the Urethra: Being the Substance of the Lectures Delivered in the Theatre of the Royal College of Surgeons, in the Year 1830, and in the West-Minster Hospital, in 1833 and 1834

**Published:** 1834-10-01

**Authors:** 


### REVIEWS.

*On the Anatomy and Diseases of the Neck of the Bladder, and of the Urethra: being the Substance of the Lectures delivered in the Theatre of the Royal College of Surgeons, in the year* 1830, *and in the Westminster Hospital, in* 1833 *and* 1834, by G. J. Guthrie, F.R.S., Surgeon to the Westminster Hospital, and to the Royal Westminster Ophthalmic Hospital, &c.—London, 1834. 8vo. pp. 284; three coloured Plates.

There are certain diseases which, either from their importance, the frequency of their occurrence, or the great relief afforded them by our art, are at all times interesting to the profession; and there are also certain authors who, from their reputation and talents, at once command public attention to their works. Among such authors Mr. Guthrie most deservedly ranks high; and the diseases of the urethra are preeminent in interest, perhaps, above all others which fall to the care of the surgeon. Hence, when the present work was announced, our expectations were raised to no ordinary degree; but we must confess that, since its perusal, they have given place to astonishment. Originality we expected from Mr. Guthrie's zealous industry and known independence of mind; but we were not prepared to find an attempt at novelty in every branch of the subject. It avails not with our author that these diseases, and the structures in which they arise, have been studied with the greatest assiduity, by the ablest surgeons and the most dexterous anatomists; their researches, in his opinion, have led only to errors, while their mis-statements are at least as numerous. On the anatomy, the physiology, and pathology of the bladder, urethra, and prostate gland, new facts are stated, and new hypotheses broached. Such a work would naturally excite attention, though written by an unknown hand; but, coming from Mr. Guthrie, it deserves, and shall receive, our most diligent and impartial consideration.

The first lecture is devoted to the *Anatomy of the Bladder*, which is described after the manner of the French anatomists, an abstract of which may be found in Sir Charles Bell's Treatise on Anatomy. Our author states that he differs from that distinguished anatomist as to the general insertion of the muscles of the ureters, which he believes to be inserted not into the posterior margin of the prostate gland, but into the uvula vesicæ. He adds, however, that the fibres of the bladder vary much with regard to the manner of their insertion into the prostate, and that some of them may follow the course mentioned by Sir C. Bell. We apprehend that there is not much difference in the statements of these two writers; for Sir C. Bell does not say that they are inserted into the posterior margin of the prostate: his words are these, "Where these columns unite, they are most fleshy, and their fibres are more intricate; then, directing their course towards the lower and backmost part of the prostate, they degenerate into tendon, and are inserted into the portion called the third lobe of the prostate."* Now, when the relative situations of the transverse lobe of the prostrate, and of the uvula vesicæ, are examined, the one will be found just anterior to the other, and so closely in contact, that, though admirably adapted for matter of disputation, it can never be of the slightest importance whether the fibres of these muscles are inserted into the one or the other. But, be that as it may, the truth can only be established by frequent and very minute dissection; and, as our attention has never been specially directed to this subject, we do not feel ourselves authorised to give any opinion.

We are, however, more competent to form a judgment on the value of a new hypothesis, brought forward by our author, as to the use of the oblique entrance of the ureters into the bladder. We quote the passage containing it in his own words:

"It is presumed that the ureters have no valves at their orifices to prevent the reflux of urine into them after it has passed into the bladder, that they enter obliquely to answer this purpose, and that the coats of the distended bladder, pressing on them in their oblique passage, prevents this reflux from taking place. It appears to me that this mechanism is for the very reverse object; and that the ureter opens on the peculiar condensed structure, in order that the orifice may be constantly patulous; and the obliquity of the passage through the coats is for the purpose of giving facility to its being pressed upon by the muscular coats of the bladder, when the viscus is in a distended state, and in order to delay, if not to prevent, the further flow of urine into it. When the bladder is contracted and empty, the urine passes readily into and gradually dilates it, until the desire for expulsion comes on, and leads to its evacuation; but a little more or a little less seems to have no influence in preventing the urine from finding its way in, the weight of urine descending from the kidney, readily overbalancing to a certain point the resisting power of the coats of the bladder. When the bladder is however distended, its coats no longer yield easily, the ureter is pressed upon by the muscular wall in its passage through it, and the further entrance of urine is in a great measure prevented. If this process be

* Bell's Anatomy, vol. iii. p. 398, sixth edition.

long continued, the ureter above this part dilates from the size of a crow's quill to that of a man's thumb, and even larger; the pelvis of the kidney also dilates, a low or chronic inflammation is induced, the secretory organs are pressed upon and partially removed, so that the kidney may become almost an empty bag separated by partitions, indicating only the former existence of its lobes. A total suppression of the secretion may under these circumstances take place at any time. The most remarkable example of the kind which has come under my observation occurred in the case of a lady, who suffered from a cancer of the uterus; the disease after a time extended towards the ureters, which at last were embraced and pressed upon by it as they entered the bladder. The lady, as this took place, began to suffer more than commonly from derangement in her urinary apparatus; the bladder was found ultimately, on passing the catheter, to contain little or no water; she fell into a state of low fever, became paralytic, afterwards comatose, and died. On examination, the ureters were found impervious at the part where they were grasped by the diseased structure; above this they were greatly enlarged, the kidneys were also dilated, and she had died paralytic and apoplectic, as all persons do in whom the suppression of the secretion is complete. The same changes take place, as I shall hereafter show, in most cases of chronic disease of the bladder; and it is a provision of nature for which this mechanism is intended, to prevent the too rapid flow of urine into the bladder, rather than to prevent a distention of the ureters, which could not, I believe, occur in this way, for some part of the bladder or urethra would yield by rupture or ulceration before the pressure on the secreting vessels of the kidney would be so great as to put a stop to their office, or to dilate the ureters. Nature can accommodate herself for several days, and in some instances for weeks, to a complete suppression of the secretion of urine; and for a very long time to a partial secretion of it. If the natural quantity usually secreted varies from two to two pints and a half in the twenty-four hours, and an obstruction takes place preventing its evacuation, the bladder will be considerably distended; but the same quantity will not be secreted during the second twenty-four hours, and still less during the third; when relief must be obtained by surgical means, if it does not occur otherwise, or great mischief will ensue. The provision of nature is therefore, as far as possible, to protect the bladder and urethra, rather than the constitution of the patient, the bladder and urethra being more susceptible of mischief than the system at large. I am more disposed to believe that the two bands on the triangular space, called the muscles of the ureters, are better fitted for keeping the part fixed, and for strengthening and raising it up when necessary, than for keeping open, and in a straight line, the channel of the ureters, which hardly stand in need of such particular apparatus to effect this object." (P. 6.)

If we understand Mr. Guthrie rightly, his opinion is, that the object of this peculiar formation is not to prevent the regurgitation of urine into the kidneys, but to obstruct its passage into the bladder, when that viscus is overdistended from disease. Several objections present themselves to this hypothesis: 1st, if this structure be not intended to prevent the return of urine into the kidneys, there is no other provided for the purpose: why, then, does it not make its way thither? 2dly. If it be intended to obstruct the passage of the urine into the over-distended bladder, it is obviously inefficient; for every one knows that, though the urine enters the bladder, under these circumstances, more slowly than ordinary, yet that it does continue to enter until the bladder or urethra gives way, or ulcerates, from pressure. 3dly. It is not usual for nature to provide two separate apparatus for the same purpose: the resistance of the muscular coat of the bladder must surely, without any refined calculation, be sufficient to counterbalance the mere weight of the urine in the ureters: where, then, is the necessity for this formation? Lastly, in the construction of the frame, provision is more frequently made for purposes of constant utility, than for the changes produced by a disease which does not affect one person in a hundred. For these reasons, we do not scruple to reject Mr. Guthrie's hypothesis as to the common use to which this structure is applied: we are nevertheless willing to concede, that, if the ureters be pressed upon, so as to prevent the exit of the urine, the same pressure will most probably impede its entrance. It appears to us that, in his search after novelty, he has overlooked the palpable and simple truth, and from a particular instance deduced a general conclusion. There is an anecdote told of a celebrated French physiologist of the present day, who, when describing the use of the rectum, stated to his hearers that it was intended to nourish the patient when circumstances prevented his being fed by the mouth.

There are one or two other parts of the above quotation which call for some comment. The author states that patients with complete suppression of the secretion of urine always die apoplectic and paralytic. We believe this generalization to have been made much too hastily. It is true that they not unfrequently die comatose; but coma is one thing, and apoplexy and paralysis are others. We would refer Mr. Guthrie and our readers to Cases 1, 2, and 4, of suppression of the secretion of urine, in the first volume of Sir Everard Home's Treatise on the Prostate Gland, where the patients died (as, according to our experience, they generally do,) with symptoms of typhoid irritative fever. We should not have noticed this inaccuracy in ordinary cases, but should have attributed it to the off-handed style of an eloquent extempore speaker, were it not that the work contains so many new facts and opinions, that we seize every internal evidence afforded by the book, as to the degree of caution exercised by the author in making his statements.

Again, in the last sentence of our quotation, he says, that "he is disposed to believe that the muscles of the ureters are better fitted for keeping the part fixed, and for strengthening and raising it up when necessary, than for keeping open, and in a straight line, the channel of the ureters, which scarcely stand in need of such particular apparatus to effect this object." We have not time to enter into the examination of the hypothesis contained in the first part of this passage, but we must confess our perfect inability to comprehend its termination. We were not aware that anybody ever asserted that the use of these muscles was to keep the parts straight, and the orifices open: we do indeed know that the very contrary idea was entertained by their discoverer, Sir C. Bell, and has been generally believed by anatomists. The following are the words of Sir C. Bell, which we should have thought it difficult to misunderstand: "The use of these muscles is to assist in the contraction of the bladder, and at the same time to *close* and support the mouths of the ureters."—"These muscles, which I have now described, guard the orifices of the urethra, by preserving the *obliquity* of the passage, and by pulling down the extremities of the urethra," &c. (Pp. 398, 399, vol. iii. of *Bell's Anatomy*, sixth edition.)

Mr. Guthrie entertains the opinion that the neck of the bladder is elastic, or partly so: we shall not suffer ourselves to enter into a discussion upon the subject, as we fear that, like that upon the middle coat of the arteries, it would be endless. We deem it right, however, to give his opinion in his own language, especially as it is accompanied by a very minute description of the direction of the muscular fibres of the bladder.

"The longitudinal muscular coat of the bladder is composed of a layer of fibres, which have in some parts little lateral connexion with each other, and are consequently capable of easy separation. The manner in which they are disposed you can see in the fresh bladder before you, as well as the points of communication between their respective fibres. If this coat be dissected off, by making an incision extending from the centre of the fore part of the prostate to the summit of the bladder, and if it be then turned outwards to the right and left, it will be seen that it passes over the prostate, and is not confined to the bladder alone. As the fibres descend from the top and sides of the summit, they sweep by the ureters, becoming more compact and firm, and in many cases some are reflected back upon the ureters, fixing and steadying them in their places. They then lie upon the sides of the prostate, being partly inserted into it and into a tendon, which they form on the fore part of the prostate, and which is usually double, one proceeding from each side. These tendons are in connexion on their upper part with the pelvic fascia, and are commonly termed the inferior ligaments of the bladder, and by the French, the pubio prostatic ligaments. In the preparation I now show you there was but one tendon in the centre, and the fibres from each side converged, and were inserted into it. My old master and friend, the late Mr. Wilson, who was perhaps the best, as he was certainly the most laborious anatomist of his early days in London, used to say that the number of those tendons was uncertain, that he had often found three, and even more; be they however more or less in number, they run on to be inserted into the pubes near its symphisis. If the attachment which the pelvic fascia, descending from the pubes, has with them, be dissected off, some fibres will often be seen arising from them a little anterior to the prostate, and running backwards and downwards in a radiated form, as you see in this preparation, to the fore part and sides of the prostate gland. These are the fibres which modern French anatomists depict in this situation, particularly Blandin; and they are, I presume, the anterior compressors of the prostate of Winslow, mentioned also by British anatomists, but not shown in their engravings of these parts, any more than those which I have been describing as belonging to the bladder, and compressing the prostate in a more marked manner. The longitudinal fibres of the bladder cannot embrace the back part of the prostate on account of the vesiculæ seminales, which would interfere with their continuance in this direction; they are, therefore, inserted into a sort of tendinous line, short of, but attached to the posterior part of the prostate. In these preparations, and in others on the table, you will see this apparent termination, which is underneath the uvula of the neck of the bladder. They do not, however, always terminate at this part; for in one preparation a strong band of muscular fibres is seen going into the prostate; and Mr. Hancock, who dissected it, was obliged to divide a portion of the gland, to show them terminating, and apparently inserted into the verumontanum with the ejaculatory ducts, which they appeared to surround." (P. 13.)

"The strong transverse and spiral cords, observable in the inner layer of the muscular coat of the bladder, you can see from the inside without any preparation, except at the neck of the bladder, where they are rarely or scarcely perceptible. These gentlemen and I have made many attempts to discover circular fibres around the neck of the bladder, but we have not succeeded satisfactorily, so as to show any of importance. In this preparation there are some transverse fibres, crossing directly over the opening from the bladder into the urethra, and they are the most marked I have seen or could discover, although the mucous membrane has been carefully dissected off in several of the preparations before you, both in the recent state, and after lying some days in spirits. I am of opinion then, that the portion of the bladder surrounding the opening into the urethra possesses but little muscular contractility, whilst it is endowed with a considerable degree of elasticity, which any one may ascertain by stretching the part. When the two muscular layers of the bladder contract, its tendons, inserted into the pubes, become with the prostate generally the fixed points, the urine is forced against the orifice of the urethra, which yields by its elasticity, and returns to its former state when the pressure is removed. I am aware that, in certain paralytic states of the bladder, the urine may be made to flow by pressing on the abdomen immediately over it, which cannot be done in a healthy person, and this would seem to imply that a muscular power was the cause of the urine being retained; although, on the other hand, the urine is retained and collected in considerable quantity in some paralytic cases without making its escape when assisted by the erect position, implying that there is also an elastic power acting at the neck of the bladder. That fibres have been described surrounding this part is undoubted, but no anatomist has dissected them in such menner as to admit of their being truly called a sphincter muscle, although that is the name given to them. It is possible that this part may be both muscular and elastic, and I am willing to take that view of it, but it is right I should tell you it has been supposed by the older anatomists, that the muscular power which prevented the flow of urine resided in those muscles which surround the membranous part of the urethra." (P. 15.)

"The term, neck of the bladder, is one which has several meanings; some anatomists confining it to one part, some to another, and even including the whole of that portion of the urethra which passes through the prostate gland. I always however understand, and shall speak of the neck of the bladder, as of the small part surrounding the very opening itself into the urethra, and which therefore is a ring, a little broader or thicker than the bladder itself. It is that portion on which the uvula is situated, the urethra being before, the bladder behind it; and the abruptness with which the opening commences, when viewed from within, appears to me to warrant the acceptation of the term, whilst the diseases which affect this part render it worthy of an accurate definition." (P. 17.)

The second lecture is upon the *Anatomy and Pathology of the Prostate Gland.* We shall omit the description of the former, as it resembles closely those which may be found in the best works on anatomy. The author objects to the term lobe being given to the transverse portion of the prostate, as in a healthy state there is no lobular appearance.

This is a matter of but little importance; so long as the form of the part is clearly understood, it may be termed either transverse portion, or third lobe, without any ill consequences. We are sorry, however, to find that Mr. Guthrie cannot leave the memory of the unfortunate Sir Everard Home, without casting his stone upon it: we had hoped that the controversy about the originality of his discoveries would, after his death, have slept in peace. Sir Everard Home was undoubtedly a very clever man, and a very great surgeon; and, if he erred somewhat on the score of manner, so as to render himself unpopular with his professional brethren, he suffered severely for his folly during his life-time, and has left an example to all future practitioners, by which they may take warning to avoid the rock on which he split.

We now arrive at some of Mr. Guthrie's new views with regard to *Disease of the Neck of the Bladder*. We quote the passage containing them.

"The prostate is applied above and to the sides of the neck of the bladder, but is generally wanting at the under part, unless the bar uniting the lateral lobes, or, as it is termed, the third lobe, is unusually large. The under portion of the neck of the bladder is not then always surrounded by the prostate, and the uvula vesicæ, the crête vesicale of the French, is not necessarily connected with its third lobe, or with any other part of it. On the fore part and sides the outer or longitudinal layer of fibres of the bladder pass over the prostate; on the under part they generally stop short of it, but sometimes perforate its under surface. The middle layer and the elastic or superadded structure appear to be attached to it, whilst the mucous coat, which possesses no peculiar properties, seems only to be connected with it through the medium of the cellular texture uniting it with the subjacent parts.

"It appears then that the following facts may be deduced from the preceding statements:

"1. That the elastic structure at the neck of the bladder may be diseased without any necessary connexion with the prostate gland.

"2. That the prostate may be diseased without any necessary connexion with the elastic structure.

"The preparation I now place before you is the most valuable one in my possession. It shows the elastic structure at the neck of the bladder diseased, without any affection of the prostate, and particularly of the third lobe. The patient passed his water with great difficulty, in consequence of the barrier formed by this unyielding structure, and died ultimately of the disease, after much suffering. This case and dissection establish in the most clear and decided manner the fact I have been endeavouring to impress upon you, viz. of a separate disease of the neck of the bladder, which has been hitherto considered as dependent on an affection of the third lobe of the prostate. To prove this has been the principal object in making all the dissections you have seen." (P. 22.)

"In this preparation of Mr. Andrews' the right lobe of the prostate is seen of more than twice its natural size; the left is a little larger than usual. When the bladder was opened, the orifice into the urethra was found dilated to the size of the end of the little finger, and perfectly round at its upper half, but this opening was nearly closed by the enlarged right lobe of the prostate, which lay in front of it, and pushed the urethra to the left, whilst it had drawn up the mucous membrane of the bladder so as to form a bar across its under part. This bar is quite membranous, and does not include the elastic structure which is not diseased, neither is that part called the third lobe, nor is there any projection into the bladder, save the bar or valve formed by its mucous membrane at the very meatus. This patient was eighty years of age, had passed his urine with much straining long previously to the last attack, which came on a few days before he died. The catheter was passed with considerable difficulty, and he sunk, at his time of life, under the irritation induced constitutionally. The bladder was very large, and but little thickened; the transverse bands on the back part are particularly isolated and strong. In the oval hollow behind the triangular space there were fifty small stones.

"In this case the disease was exactly the reverse of the other; the prostate was alone affected, and the bar formed at the neck of the bladder consisted of its mucous membrane, elevated and drawn tight across the under part of the opening, in consequence of its connexion with the prostate through the subjacent parts. If the prostate could have been removed, the mucous membrane forming the bar would have fallen back into its proper place. If this bar could have been divided, a great obstruction to the flow of urine would have been removed, and a proportionate relief obtained. When there is a third lobe of the prostate, and it is diseased and projects into the bladder, the elastic structure of this part usually partakes of the evil, forms a hard firm bank in addition to the nipple-like valve, and between them the retention of urine may become complete. This disease, with its complication, is much less curable than the disease of the neck of the bladder alone, and the necessity for a distinction between them is so much the greater, believing, as I do, that relief from this latter complaint is always in the hands of the surgeon.

"In its simple or first stage, when there is only a defect of elasticity, it gives rise to stricture at the very neck or orifice of the bladder, curable by common means, if properly applied. In its second stage, when the bar is formed, and becomes more or less rigid, a small bougie rests against it, and if made of soft materials bends, and cannot be made to proceed; if a solid instrument, it passes in one of the hollows on each side of the white central line, which are also deepened by the elevation of the uvula vesicæ, catches on the valve at the entrance, and, when the handle of the instrument is depressed, it raises it, bladder, rectum and all, upon its point, until the pain or the resistance induces the surgeon to forego the depression, or the valve yields or is torn, when it finds its way into the bladder; or perhaps the surgeon, not possessing much experience, is satisfied with the distance the instrument has gone in, and supposes he has passed it into the bladder. This is one of the evils which arises from the attention which has been paid to the length of the urethra with regard to inches only; for, when a man is told that the urethra is often only eight inches long, and finds his instrument has passed perhaps more than nine, he may deceive himself, although his patient is not relieved. I had a gentleman from America under my care lately under these circumstances. He had never passed his bougie beyond the neck of the bladder, although he and his surgeon supposed they had done so. When I succeeded in doing it, he became sensible of the difference; and I desired him, on his taking leave, always to use in future a No. 12 catheter, with a very round point, that the passage of urine through it might convince him of the fact." (P. 24.)

We shall make no observation on his views for the present, as a better opportunity will present itself, in another part of this review.

The author next adverts to the *Formation of the Pouches in the Bladder*, by the protrusion of the mucous membrane between the fibres of the muscular coat. As to the manner in which they arise, all surgeons, we believe, agree, viz. from obstruction to the free passage of the urine under ordinary efforts, and the consequent violent contraction of the bladder. Mr. Guthrie, however, appears to limit them only to cases of disease of the neck of the bladder. If this be his intention, we are certain that he is mistaken, for, within the last few months, we have had the opportunity of examining the body of a patient who died of disease of the kidney, in whom both the neck of the bladder and prostate were, as far as we could discover, perfectly healthy, but who had an extremely small stricture just anterior to the bulb: nevertheless, there were three distinct pouches, and one of them of considerable size.

Our author attributes what he terms the fluttering blows of the bladder, to the sudden descent of these pouches upon the catheter. He states that he formerly believed that "they depended upon some unusual action of the oval cavity of the fundus, or of the base of the triangular space, acted upon irregularly by their own fibres, and by those passing perpendicularly and across by the sides of the neck of the bladder, and which have been considered as a sphincter."

His opinion was changed by examining the body of a patient in whom this particular blow occurred, and discovering five pouches in his bladder.

We must confess that, in spite of this case, we are more inclined to believe in his former hypothesis than in the latter, for we have met with this blow, not long since, in a case where we have not the slightest suspicion of the existence of pouches.

We have only to turn over two pages, and we find another, and still greater novelty; no less a one than the discovery of the prostate in the female!

"It is usually said that the female has not a prostate, but merely an erectile tissue surrounding the neck of the bladder, to which I do not assent. If the word prostate be used with reference to its derivation, as standing before the vesiculæ seminales, certainly a woman has not a prostate, because she has no vesiculæ seminales, but if it be used as a substantive word, to express a particular thing, in the same manner as the words arteria innominata are now used as a name for a particular artery, which formerly had no name, then a female has a prostate, for there is a substance of the same shape, form, and nearly of a similar structure, surrounding the commencement of her urethra. It is the size of the prostate in a boy before the age of puberty, and resembles very nearly in external appearance the same part in the male. Here are the prostate and bladder of a boy of twelve years of age; and these two other preparations in spirits show the bladder and prostate in the adult female. One bladder is opened from the fore part, the other from the back part, to show how much of this substance lies before and behind the commencement of the urethra. You will observe, in these two recent dissections of the same parts, that the band, on which the ureters are situated, is less marked, as well as those descending from them, than in the male, but the elastic structure is equally evident. The ejaculatory ducts of the male, opening into the urethra, are of course wanting, and there does not appear either to be any ducts of the prostate, so that, perhaps, this substance may be considered in the female to be partly destitute of the follicular or glandular structure, which gives the additional bulk to the male. The fibres of the bladder have the same arrangement in the female as in the male, and I am therefore induced to believe that the prostate gland in the male has at least three offices, viz. 1, to stand before the orifice of the bladder, and give it and the urethra which it surrounds support, and a point more or less fixed, upon which it may act in expelling urine; 2, to secrete a fluid peculiar to itself; and, 3, to receive the ducts conveying secretions from other parts; which two latter uses I do not attribute to it in the female, and the want of which may account for the difference of size in this part in the two sexes. At all events, it shall, gentlemen, be our will and pleasure, until further orders from the critics, to give the ladies an organ which they have not of late years been supposed to possess; but, whilst we call it the female prostate, we will hesitate in adding the word gland. Cowper, who was well acquainted with it, calls it corpus globosum." (P. 35.)

We feel scarcely inclined to comment upon this: we might safely leave it to our readers, were it not that our respect for Mr. Guthrie demands a careful examination of his opinions. He sets out by stating that the prostate, though named originally from its situation, must be now considered "to be a substantive word, and to mean a particular thing." He then goes on to inform us that its structure in the female is "nearly similar" to that in the male, (only allowing for difference between erectile and glandular tissue); that it does not secrete a fluid, and that it does not receive ducts conveying secretions from other parts.

Now, without venturing to anticipate the orders which will be received from the various critical synods sitting in this realm, we enter our protest against the new name; for dissimilarity of function overpowers the similarity of situation, and forbids our allowing of the identity of the prostate and the corpus globosum. The author would not term the bladder in the male a uterus, because it is anterior to the rectum: we ask him only to apply the same rule to the prostate.

The third lecture is devoted to the *Demonstration of certain New Muscles* which Mr. Guthrie has discovered. He states that he could never get a clear view of Mr. Wilson's muscle; yet we have seen it many times very satisfactorily dissected. In place of the old one, however, he demonstrates some new muscles; of which he gives the following account.

"The two preparations I place before you now are the most perfect. One shows the membranous part of the urethra surrounded by a well-defined muscle. On the upper part there is a central median line of tendon, which runs backwards to be inserted into the fascia covering the upper surface of the prostate; and again forwards on the urethra through the triangular ligament, to be inserted in front of it near the union of the corpora cavernosa. On the under part a similar tendinous line is to be observed, which is attached backwards to the fascia underneath the apex of the prostate, and forwards to the central tendinous point in the perinæum. The muscle on its upper surface is covered by fascia descending from the pubes which adheres to it, and this I take to be what Mr. Wilson described as the tendinous origin of his muscle, and from which he supposed the fibres descended to surround the urethra, which they really do not. From the median tendinous line in the upper part of the urethra the fibres pass outwards on each side, converging towards the centre, where they form a leg, as I

term it, of muscular fibres. On the under surface the same thing takes place; and a leg on each side being thus formed, from the superior and inferior fibres running from each half of the urethra, they pass outwardly, that is, transversely across the perinæum, to be inserted into the ascending ramus of the ischium a little below its junction with the descending ramus of the pubis on each side. These attachments are cut off in this preparation, but, when I pull on each of these legs, you see that they surround the urethra like a sling.

"In the preparation I now present to you nearly all the parts are in situ. You see the muscle or muscles and their origins one on each side, which are very peculiar. They are inclosed between the two layers of fascia, forming what is commonly called the deep perinæal fascia. The anterior layer is turned down as well as the triangular ligament from the pubes; and you see the muscle on each side with its origin, and lying between the anterior and posterior layers of the fascia. The pudic artery on the right side runs in front of it, and you see here the division of that vessel into the two arteries of the corpus cavernosum and of the dorsum of the penis. Cowper's gland on each side lies under or posterior to the muscle, and seems to be enveloped by it. The inner layer of fascia passing on the inside of the muscle reaches the inner edge of the levator ani, round which it turns to invest the urethra and prostate; so that this fascia separates these two muscles, the fibres of which also run in different directions. This is distinctly shown; and it is manifest that the muscle I have described is inclosed between the layers of fascia, which is, I presume, the reason why it has escaped the observation of the very many minute anatomists who have investigated these parts.

"The preparation I now show you is an internal view of the pubes, with its descending rami, and the ascending rami of the ischium. The deep fascia of the perinæum is preserved: the urethra is seen passing through the triangular ligament, surrounded before it passes by the muscles in question; the internal layer of fascia is turned down, and the two legs of the muscles are seen passing outwardly or transversely to their origin.

"In the two recent dissections I now show you, the same muscle or muscles are seen in the female, proving therefore by their existence that they are not sexual muscles, and that the opinions of Mr. Wilson and Sir Everard Home, who believed them to be so, were erroneous, whilst there can be no doubt of their being intimately and essentially connected with the due retention and transmission of the urine. Taken as a single muscle, it has then two origins and five insertions, or six, if the superior attachment of Mr. Wilson be considered double. When it acts, which it must do from its origins from the rami of the pubes, it can compress the urethra so as to close it, I conceive, completely after the manner of a sphincter; whilst, from the attachments of the muscles, from their origin and being inclosed between the layers of fascia, they must also draw it towards the pubes. This muscle has a singular resemblance to the accelerator urinæ, situated outside the fascia, and is capable of acting, I am led to conclude, with great energy. The preparations are preserved in the Museum at the Ophthalmic Hospital, Chandos street, Charing cross." (P. 40.)

This description is followed by various woodcuts, all representing this muscle as clearly as possible; so that we wonder at our own and our predecessors' carelessness in suffering them to escape observation. As we have never seen the muscles, we can offer no opinion as to the accuracy of the drawings.

The fourth lecture commences with a very minute *Description of the Acceleratores Urinæ Muscles,* for which, unfortunately, we have not space: we rather prefer presenting our readers with the author's observations on the length of the urethra, as of more practical importance.

"There is one urethra on the table eleven inches long, and another eight, and several of intermediate lengths, depending principally, it is true, on the pendulous portion for the difference, but not entirely; for the age and stature of a person, even after he has become an adult, often makes an essential difference when compared with another in those parts which are more fixed. I place no reliance whatever on the measurement of the urethra made after death, for, although the urethra may be then eleven inches long, as in the preparation before you, I never met with a case, unless there was a diseased prostate, in which a catheter ten inches long was required to draw water: on the contrary, one eight inches long will generally be found sufficient, and I have known one of seven answer well; the additional inches being usually gained by the elongation or stretching of the parts during life or after death; and if a surgeon calculates inches as his instrument proceeds, instead of considering the points of attachment as so many land-marks to guide his progress, he will be frequently in error, and always liable to do mischief. I believe the mistake of most consequence takes place in regard to those two parts which are called membranous and prostatic. In the preparation before you, in which the urethra is eleven inches long, the length of these two portions is near three inches. From the orifice of the bladder to the triangular ligament, the usual length of the prostatic portion is estimated at about fifteen lines, the membranous at about twelve, or something more than an inch for the prostatic portion, and perhaps a little less than an inch for the membranous. In this very urethra, eleven inches long, I have no doubt but a catheter nine inches in length would easily have drawn off the urine during life. I am satisfied that one of eight inches would have done it, and the membranous and prostatic portions, which now appear to be near three inches in length, would have been cleared by an instrument very little more than two; and this would occur from the manner in which these parts are supported and maintained against the pubes by the fasciæ, which attach and connect them and the surrounding parts to each other. When the prostate is diseased, on the contrary, the urethra at this part is made to undergo a considerable degree of elongation, and a catheter, under such circumstances, should be longer and larger in the curve.

"The length of the spongy portion of the urethra must always be uncertain, and is of no consequence. The orifice of the urethra when in a normal state is always vertical, and closed, the sides being applied to each other." (P. 52.)

There are some other very good observations on the size of the urethra and its orifice. When the latter is small, either from natural formation, or from a bridle passing across a portion of it, Mr. Guthrie generally divides it with the knife. This plan has lately been much preferred by surgeons to the use of the conical dilator, which not only occasions more pain at the time, but is frequently followed by inflammation of the part, which renders the process of dilatation extremely slow. The conclusion of the lecture contains a description of the various solid instruments now in use, and the method of employing them.

In the fifth lecture he dwells at considerable length on the *various Curvatures requisite in particular Cases of Disease of the Prostate.* His observations on this subject are practical and exceedingly good; but, as they occupy too large a space for quotation, and will not admit of abridgment, we must omit them, but not without great regret.

The sixth lecture sets out with a declaration that appears to us rather extraordinary, viz. "that it is a fact acknowledged by almost all surgeons of experience, that the prostatic and membranous parts of the urethra are not usually the seat of stricture." We are unwilling to place our own experience in opposition to Mr. Guthrie's, but we have always heard, and have indeed found, that the majority of strictures are to be met with in the membranous portion. Sir Benjamin Brodie, in his excellent work on the diseases of the urinary organs, states that "the ordinary situation of a permanent stricture is at the anterior part of the membranous portion of the urethra, just behind the corpus spongiosum," page 6. Mr. Wilson also says, "the part where strictures most usually begin is where the bulb unites with the membranous parts of the urethra," page 367.—*(Wilson on the Urinary and Genital Organs.)* Sir E. Home considers the most common situation of stricture to be about seven inches from the orifice. It is true that Sir Astley Cooper believes them to be most frequent just anteriorly to the bulb, but he places next in order those in the membranous portion. Surely Mr. Guthrie will not refuse to these authors the title of experienced surgeons; and, if he does not, we are at a loss to reconcile his statement with the fact.

This error is not of slight importance, as the author founds upon it his principal argument to prove that the spasmodic contraction of muscular fibre is not the original cause of permanent stricture. As matters stand at present, we are inclined to believe that both sides of the question have some share of truth in them. When stricture occurs in the membranous portion of the urethra, the spasmodic contraction of the compressor urethra muscle gives rise to inflammation, which occasions the thickening of the parietes, and the consequent diminution of the size of the canal. We are borne out in this opinion by the fact, that all strictures in this situation are remarkably liable to spasm, both in the onset and progress of the disease. On the other hand, as there is no compressor urethræ surrounding the anterior part of the canal, we think Mr. Guthrie's solution, which we quote, may be applicable here.

"The whole anterior portion of the urethra, or that part in which stricture is usually situated, is surrounded by the corpus spongiosum; and it is to it that I am disposed to attribute the principal share in the formation of the worst kinds of permanent stricture, and the great difficulty which is experienced in effecting a perfect or radical cure. When the membranous portion of the urethra passes through the triangular ligament it preserves its fibro-cellular exterior, and more particularly at the upper part to which the bulb of the corpus spongiosum is not applied; but, when the corpus spongiosum does surround the urethra, this external fibrous cellular covering leaves the internal mucous membrane, and is attached to, and merges in the elastic structure of the internal layer of the erectile tissue of the spongy body; to which the mucous membrane is so intimately attached as to be separated only by scraping with considerable care; and thus destroying the shreds of attachment between them, which are in some places more strongly marked in the general cellular attachment than at others." (P. 75.)

"The whole corpus spongiosum, including the bulb, seems to be formed originally in two symmetrical halves or parts, and to unite to form one body, much after the manner of the corpora cavernosa penis. * * *

"The elasticity of the urethra when surrounded by the corpus spongiosum, is best shown by introducing a solid sound into it, when, by turning the point downwards, it may be stretched to a considerable extent in every direction. This may be done in the living body without giving pain, with the exception of the orifice of the urethra, which will yield but little. If the person should have an old narrow permanent stricture at the distance of three inches from the orifice the experiment is made most conclusively, for the urethra stretches at every part anterior to it with great ease; but, when the solid sound reaches that point it can penetrate no further, the elasticity of the part is lost, the hardened obstacle formed by the stricture is distinctly felt from the outside; and, by a little turning downwards of the sound, its point can be felt through the external parts, projecting below the obstacle, and carrying the urethra before it. If a sound just large enough to go through a stricture of this kind is passed, and the part is examined between the finger and thumb, the extent of the stricture may be easily ascertained by the hardness, which is quite peculiar and distinct from the natural structure of the part either before or behind it. If the instrument is withdrawn, and the same spot is again examined, the hardness will be very perceptible, when compared with the soft elastic sensation communicated by the spongy body in its natural state. The hardness is sometimes like a cord, and occasionally, when circumscribed, like a small hazel nut. In the erectile state this hardened part is not augmented in size, although the spongy body is distended before and behind it; whilst it remains a stationary hard line or spot, connecting the two distended parts together, and, when the stricture is in an irritable state, often giving pain. If this hard part be cut into, the corpus spongiosum seems to have lost its spongy appearance, its erectile texture has become consolidated, and resembles rather a solid gristly substance than an elastic structure. This kind of disease is very apt to form when the urethra is ruptured, during the severity of what is termed a chordee. It yields to the distending power of the two erectile bodies, and the inflamed part which has lost its elasticity is torn; the tear extends into the spongy body itself, blood flows freely from the orifice of the urethra, and the cells of the corpus spongiosum around the rupture become loaded with it. Inflammation follows, and without great care be taken in the treatment, a permanent stricture is the result." (P. 77.)

The next passage which we present to our readers, is not intended to teach them any new surgical truths, but it is rather to discharge a debt which we owe to the author. He confesses himself to be at a loss; and, as we have learnt much from him, we think it only fair that he should learn something from us.

"When the mucous membrane is inflamed, and has lost part of its elasticity, it does not always yield as readily under distension as some of the interstitial parts of the corpus spongiosum; which, when they give way, allow the blood to be effused in the strict sense of the word; and a soft swelling takes place, which is a sufficiently remarkable, although not a very common accident. I have just now under my care a young gentleman, who has a soft swelling of this kind, about two inches and a half from the orifice of the urethra, and which appeared suddenly. The urethra was inflamed at the time, but was not ruptured; a full-sized bougie could and can be readily passed along it. It gradually altered its appearance, became less, and would I think have gone away altogether, had not another gonorrhœa supervened, which, by adding new symptoms, has rather increased than diminished it, and without great care a stricture will possibly be the result. In a case which I treated many years ago, in the York Hospital, the swelling situated in the same place was as hard and as circumscribed as if a Barcelona nut had been inserted into the under part of the urethra. It was quite cartilaginous to the touch, and the man made his water almost by drops. I removed this disease by the repeated but careful application of the argentum nitratum, so that no signs of it remained externally, the hardness having gradually diminished until it went entirely away. The man, a soldier, was to have been discharged, but, on leaning over his bed to fold up the blankets one morning, he fell forward dead. I opened him next day, and found his heart diseased. The urethra appeared quite sound, and, to my great surprise, nearly as much so at the part which had been affected as any other. I had a preparation made of it, and it is or ought to be in the museum at Chatham. Lest you should go away with the impression that a stricture of this kind may always be cured by caustic, I must mention to you the case of a gentleman who consulted me a short time afterwards. He had a similar swelling, situated at the part where the scrotum joins the penis. I was delighted to have the case, and felt assured of a similar termination, but no such thing took place: the swelling and hardness increased rather than diminished, and at last I was obliged to divide the part from without inwards to save his life, by giving free passage to his water. I have since had many other cases of a like nature, all of which have been treated and relieved with various degrees of success, but none so pre-eminently well as the first. You will ask me, perhaps, why? I can only say, it is as difficult to answer you on this point, as it is to tell you why, in some cases of almost impermeable stricture a permanent cure is effected by simple dilatation, whilst in others, as nearly alike as possible, the relief obtained is only temporary. It depends on the various shades of distinction between diseases, and on the particular extent to which each peculiar structure is affected. Experience assisted by careful observation may enable us to select the best and least dangerous mode of treatment, but it has not as yet enabled us to mark all the distinctions and shades of difference between these diseases, which it is necessary we should know, to arrive at more perfect knowledge of their treatment." (P. 79.)

The author has here, without being aware of it, described two perfectly different diseases. In the first two cases there was no stricture, in the ordinary sense of the term; for, in the first, he tells us, that a full-sized bougie would pass, and he was fortunate enough to have an opportunity of examining the other, and no trace of stricture was to be discovered. In the last case, though the circumstance is not mentioned particularly, we have no doubt that a stricture existed. The former disease may be termed inflammation of a mucous follicle in the urethra, and it generally occurs within three inches of the orifice. The author's description of it is so accurate that it cannot be mistaken, for it feels exactly like a Barcelona nut imbedded in the corpus spongiosum. It generally subsides of itself, but it may always be readily cured by the application of a leech or two, rubbing the under surface of the penis with mercurial ointment combined with camphor, and directing the patient, when he passes water, to press with his finger slightly on the tumour, so as to prevent the accumulation of urine at the part. If in a few cases it be more obstinate than usual, a small gum catheter may be introduced into the bladder, and left there till the cure shall have been completed. We would earnestly caution our readers against the employment of the caustic in these cases, as it is a most efficacious means of exciting suppuration in the tumour. The other disease described by the author is of a much more serious nature. It generally occurs in cases of long standing, and is only to be met with at the part named by our author, viz. where the stricture joins the penis. In its nature it is analogous to the pouches formed in the bladder. The patient has a stricture for a long time, and the water is constantly impeded at this part; the mucous membrane of the urethra is dilated by the pressure, and at last a sinus is formed, with its orifice posterior to the stricture, but, passing in a direction forwards, so that the tumour appears rather in front of the contraction of the canal. Inflammation taking place around the sinus causes a deposit of lymph; hence the induration. A very different prognosis should be given in these cases; the stricture may be dilated, but the sinus is not always cured. The best method of effecting this is to keep a full-sized catheter in the bladder after the stricture is dilated, while tolerably firm pressure is applied to the tumour; the unguent. hydr. c̄ camphorâ may be rubbed in as before. We trust that we have made this subject intelligible, not only to the acute understanding of the author, but to all our readers. It is but justice to Mr. Guthrie to state, that if he was not himself aware of the nature of these diseases, he knew at least the method by which that information was to be acquired, viz. from "experience, assisted by careful observation." In confirmation of his opinion we have only to state, that for the description of the latter disease we are indebted to Sir Benjamin Brodie.

At the commencement of the seventh lecture, the author enters into the discussion of the muscularity of the urethra, and embraces the negative opinion: we have not, however, sufficient space to follow him, as we must confine our attention to more practical subjects.

The next instance in which Mr. Guthrie differs from established authorities, is in the application of the terms spasmodic stricture, and spasm of the urethra.

"The more common cases which are usually considered spasmodic, are those of young men, who, when suffering from gleet or gonorrhœa, imperfectly or only partially cured, are tempted to commit an excess in wine or punch. After sitting some time, they feel a desire to make water, which they repress, or perhaps indulge with some difficulty, but which increases, and is soon found to be irrelievable without assistance. The greater the effort, the more determined the straining, the greater the agony; and the sufferer, with despair depicted in his countenance, entreats relief." (P. 89.)

"These are called cases of spasm,—I call them cases of inflammation,—and which induces a want of consent, as Sir C. Bell expresses it, between the muscles of the part, so that, when the bladder acts, the muscles surrounding the urethra will not act by yielding and dilating as they ought to do, but remain, or become more permanently contracted: the urine is forced against the inflamed and contracted part of the urethra, and by its irritation increases the mischief." (P. 90.)

The author acknowledges in an adjoining passage the utility of opium in the relief of these cases, and it is therefore clear that his distinction is one of nomenclature, rather than of practice. A want of consent in the action of muscles is the same thing as spasm; for the muscle which refuses to be relaxed with its fellows, is in a state of spasmodic contraction. Mr. Guthrie recommends, in these cases, the immediate use of instruments; and would employ leeches, bleeding, the warm bath, James's powder, and opium, as subsidiaries.

The following account of the effects of over-distending a stricture is extremely good, and is not generally known, or, if known, not attended to sufficiently.

"A gentleman presents himself with a stricture, at two, three, four, or more inches in the canal, which at the orifice is capable of admitting a No. 13 or 14 solid bougie, but the stricture will only allow a No. 6 to pass. You dilate this slowly until a No. 10 or 11 will pass easily, when, anxious to have his case completed, your patient presses you to increase the size, and, yielding to his solicitations, or tempted by your own desires, you pass over the intermediate number, and take the 13 or 14 at once: you will often be able to succeed, with little uneasiness at the moment; but your patient, on wanting to make water, finds he cannot do it: he strains, but it comes only by drops. The desire increases to misery, and he sends for you. Now, what would you do? Theory teaches, put him in the warm bath, give him an opiate, bleed him if necessary, for the case is one of inflammation; but practical surgery says, do nothing of the kind, but take a small elastic gum bougie without a stilet, and draw off the water. Your patient will be immediately relieved, will wish you good night, if he is a wise man, and go to sleep: when he wakes, he will make his water without your assistance; but, if you try to pass a bougie for him some six or eight days afterwards, you will find yourself very much where you were when you began, that is, able to introduce only a No. 6. The part has contracted as much as ever, although perhaps it may be more readily dilated than it was before. The necessity for great gentleness in all these cases cannot be more forcibly exemplified." (P. 95.)

The eighth lecture contains an excellent account of the symptoms of permanent stricture, and of the best means of conducting an examination of the urethra. But, as our quotations have been already very numerous, and as there still remain several novel opinions to be considered, we must deny ourselves the pleasure of presenting our readers with any portion of it. The author here sustains his high character as a practical surgeon; he supplies us with most minute description of the bougies to be employed in each particular case. We rather suspect that he thinks too highly of the dilator recommended by Mr. Arnott, (to the partial invention of which the author lays some claim,) for, as far as our experience goes, we should say that, when the urethra will permit the passage of the dilator, the cure may be easily completed by the ordinary instruments.

The author regards the nitrate of silver as a useful application in certain cases of stricture, which he does not exactly distinguish, and, on the whole, may be considered more favorable to its use than most modern surgeons. In discussing the subject he introduces the following excellent observations on hemorrhage from the urethra.

" The hemorrhages from the urethra are caused by the sloughs separating, and leaving the cells of the corpus spongiosum exposed, or by the ulcerative process extending to some small vessel, the canal of which is partially opened. These, it is said, cease of themselves, although not until a great loss of blood has been frequently sustained, and it has been recommended to let the parts alone. I cannot give you any opinion formed from personal experience, as I have never seen one of these bleedings from caustic; but I conceive that they should be met and treated like hemorrhages from the same place from other causes, which appear to me to be of a similar nature.

"The most alarming hemorrhages I have met with have been from common causes; and I will mention to you two of them of the most prominent kind, as they also point out the practice to be pursued in such cases. The first occurred in a gentleman living in Cockspur street, who had had a catheter passed by a surgeon of great reputation and ability in the morning, without either pain or inconvenience. On his return home, he found there was a considerable oozing of blood, which continued during the day, and induced him to send in the evening for his surgeon, who was unluckily out of town: the bleeding increased in the night, and in the morning early I saw him. There were several tubs of ice and water in the room, all apparently containing a considerable quantity of blood: his face was deadly pale, the pulse scarcely perceptible, and he said he had bled a pailful, which was of course an exaggeration. The bleeding was arrested in a few minutes, by pressure applied in the proper place, (on the perineum,) and did not return.

"The second occurred in the case of a tradesman, who had passed a common soft bougie for himself, the point of which had caught on some small opening, and, it is presumed, had penetrated into it: he bled for two days and two nights, when I was desired to see him, in Paddington street. I found him kneeling in bed, and straining violently to pass his water, but which came with great difficulty, as the bladder contained a good deal of coagulated blood, which had passed backwards into it. He was as white as a sheet, and fell back in his bed, nearly insensible, almost as soon as I entered the room, having, as he said afterwards, passed several quarts of what (as it all coagulated) he considered to be pure blood. As urine and blood coagulate together, when out of the body, in equal proportions, it is probable that only half of it was blood. This bleeding was also arrested in a few minutes by pressure, and did not return.

"For the purpose of knowing where to make the pressure, any light, flat, and narrow, but firm substance, should be prepared, such as a piece of cork, which can always be procured. The patient should then force all the coagulated blood out of the urethra; and, as the bleeding usually takes place in these cases from that part which is anterior to the triangular ligament, pressure can readily be made upon it externally; but, as it may be made a little before or behind the exact spot, in either of which cases it would be useless, the selection of that spot must be well made. This is done by beginning as far back as possible, and gradually bringing forward the finger by which the pressure is made. At a certain point the flow or dripping of blood will be arrested, and the precise spot from which it comes will be in all probability a little behind where the finger rests; a fact which can also be easily ascertained by carrying the finger a little backwards, when the blood will again flow. The bit of cork or pad can now be duly placed, and the patient should be desired to make pressure on it himself, and which he can often more readily do than an assistant.

"When the hemorrhage comes from the prostatic part of the urethra or neck of the bladder, cold water, rest, and an opiate, will suffice to stop it, provided it has been caused by some accidental circumstance, and does not arise from disease of a fungous or malignant nature; in which cases, nothing can prevent its return, or even its continuance." (P. 135.)

The author afterwards separately mentions the occurrence of hemorrhage from fungoid disease, and relates some interesting cases of these affections.

The next lecture, which is upon the treatment of retention of urine, commences with the recommendation to employ the bougie early in every case of what is termed spasmodic stricture, and, when this fails, to have recourse to other instruments. He speaks highly of the use of a very fine catgut bougie, a plan of treatment which he had been for some time in the habit of employing with the greatest success. It appears indeed to us that almost every case may be relieved by the dexterous use of this instrument, and it is attended with this advantage, that, if it fails, it does not (as the larger ones do,) increase the irritation previously existing, and other instruments may afterwards be advantageously employed. When, however, all instruments fail, recourse must be had to general remedies, such as bleeding, opium, &c. But if these also fail, there remains no resource but an operation. Mr. Guthrie prefers that of cutting down upon the urethra; and we think that he certainly chooses the best of the operations. Nevertheless, it must be remembered, that it is attended with great difficulty in the performance, and that, unless the surgeon be well acquainted with the anatomy of the parts, and moreover is in the habit of operating, he will probably not succeed in accomplishing his object. For this reason we believe that the operation of puncturing the bladder by the rectum will continue to be more generally employed, though we should recommend all surgeons to endeavour to master the former, as it is otherwise greatly preferable. We quote the author's description of his mode of performing the operation.

"The improvements which have been made of late years in the practice of surgery render this operation less necessary than formerly: cutting out, or cutting into, portions of the urethra, which

I have seen attempted, are, like cutting out testes, comparatively obsolete operations; but still an opening into the urethra may occasionally be required, and, when the disease is situated at the termination of the bulbous portion of the urethra, or even further back, I recommend the operation to be done in the following manner, as much the most simple and certain. The patient being placed and secured as in the operation for the stone, a catheter or sound is to be passed down to the stricture, and held steadily against it, the concavity being as usual upwards, the point directly applied to it. The rectum having been previously cleared by an enema, the forefinger of the left hand, being duly oiled, is to be introduced into it, and the membranous part of the urethra and the prostate are to be examined, as well as the bladder; the state of which will in all probability have been previously investigated. If the membranous portion of the urethra is dilated by the urine, so much the better; but the object of introducing the forefinger is to ascertain the relative situation of the upper part of the rectum and the urethra, which latter part only touches, or is nearly in direct application to the rectum, at the termination of its membranous part and the commencement of its prostatic portion. There is a certain distance, which is greater or less in different individuals, between the last inch of the rectum and the urethra placed above it. The two parts form two sides of a triangle, the apex of which is the prostate, the base the external skin. It is within the two lines of the triangle that the operation is to be done. The surgeon, taking the catheter in his right hand, whilst the forefinger of the left is applied to the upper surface of the rectum, moves the point upwards and downwards, so as to communicate with the forefinger of the left hand, and to convey to it a knowledge of the situation of the extremity of the instrument, and particularly of the distance between them, which the motions given to the catheter by the right hand will clearly indicate. The thickness of the parts between the obstruction and the rectum can be estimated with sufficient accuracy, both at the point where the left forefinger is applied and at the surface of the skin; for, although the membranous part of the urethra cannot be easily felt from an incision made on the left side of the perineum, it can always be distinguished from the rectum. The next step of the operation is to divide the skin, cellular membrane, fascia, muscular and tendinous fibres, which may intervene between the upper surface of the rectum and the under surface of the anterior and middle portions of the membranous part of the urethra. This is to be done by a straight, blunt-backed, narrow, sharp-pointed bistoury, fixed in its handle; and there are two ways of commencing the operation: the first, when the obstacle is behind the bulb, and the external parts are not diseased, may be done by a straight incision, in a perpendicular direction; in which manner the operation may always be done, if the surgeon is well acquainted with the anatomy of the parts; but, if he is not, or they are very much hardened, and consequently unyielding, a transverse, curved, or concentric incision, should be made across the perineum. This gives room, and allows the parts to be separated as much as they will admit. If the transverse incision is not adopted, the point of the straight bistoury is to be placed on the skin a little above the verge of the anus, the cutting edge being above, the blunt back towards the rectum, the handle being a little depressed, the point a little inclined upwards. The degree of inclination necessary to carry the knife inwards for the distance of an inch, and clear of the rectum, will be indicated by the finger in that part, and the eye of the operator will correspond with the point of the forefinger, so that the bistoury may be steadily pressed in to that extent, and then carried upwards, and brought out in the exact median line, making an external incision of at least an inch and a quarter to an inch and a half, as regards the external parts; and which may be then extended, as space is wanted for the prosecution of the operation. The part being sponged, the surgeon again introduces the bistoury in the median line, the point being directed upwards and backwards towards the urethra, and he may then deepen the cut. The forefinger in the rectum will always tell him where the back, and consequently where the point, of the bistoury is. The opening now will be sufficiently large to allow the operator to lay aside the knife, and to feel for the urethra with the point of the forefinger of the right hand; an assistant keeping the catheter steadily against the stricture, the end of which will now be readily felt. If the point of the forefinger of the right hand does not go beyond it, and touch the sound part of the urethra, which is dilated by the urine in the generality of cases, the knife is to be resumed, and the forefinger, being withdrawn from the inside of the rectum, is to be placed in the wound, on the outside of the rectum, which is to be depressed as much as possible; the back of the knife is then to be turned to it, whilst the point exposes and opens the urethra, and which it can do very easily near the apex or transverse portion of the prostate, or at the termination of the membranous part of the urethra; but it is not necessary to go so far back, and the membranous portion may be opened at its middle with every advantage, and with perfect safety to the gut. A good anatomist and surgeon will open the urethra in this way sooner than the mode of doing it can be described; the urine will make its escape, and the patient will be at once relieved. Whether the stricture shall be now divided or not, is a question presently to be considered; but the cure can be completed either with or without it." (P. 166.)

Further on the author states that he is an advocate for the division of the stricture. We acknowledge that we think this step unnecessary; but, as we do not see any particular disadvantage attending it, we leave it to the judgment of our readers. When the pressure from the distension of the bladder is taken off, the stricture very readily dilates under the use of the bougie. Should the urethra give way before assistance arrives, the author agrees with all good surgeons, that free and deep incisions offer the only chance of saving life.

The twelfth lecture treats of impassable stricture, and its treatment. While Dupuytren was putting in force his plan of exhausting the irritability of the urethra by long-continued pressure, the author was conducting a series of experiments upon the same subject in this country. The following is Mr. Guthrie's method of proceeding.

"The best dilating material is a hollow gum elastic bougie, of a medium size, perfectly smooth, and tolerably round at the point, so that it may give as little uneasiness as possible. A very small bougie gives more annoyance than a larger one, is retained with more difficulty, and is more likely to give rise to irritation, in which case it should be removed, and, after a little delay, replaced by a larger one. If this should also give rise to irritation, which rarely occurs, it should also be removed, and the irritation subdued by warm fomentations, by opiates, and perhaps by the application of a few leeches. There are very few cases which require any thing more, provided the patient will be perfectly quiet, live moderately, and preserve the recumbent position, until the irritation has subsided, when he may sit up and walk about his room in his ordinary manner. The bougie is to be fixed in the urethra in the same way as a gum elastic catheter is fixed in the bladder; it should project about one inch beyond the orifice of the urethra, and rather less than more. The point should press against, or rest upon, the stricture with the greatest possible gentleness, so that it may not give rise to inflammation or to ulceration, and yet should press just so much as to cause absorption. It is an admitted point in the animal economy, that new-formed parts, whether laid down in reparation or in disease, do not resist a stimulus in the same manner as parts of original formation. They are in fact removed by the action of the absorbents under the application of a stimulus, which has little or no influence on those parts which have undergone no change, and are coeval with the existence of the individual. The pressure made by the point of the bougie, and which ought always to be an elastic one, should therefore be nicely regulated, so that it may do this, and no more. It is a point which requires attention, and some little experience, although a due knowledge of it is soon acquired. It should never be so great as to give pain, or indeed uneasiness, and yet it should be continual. The patient readily learns what is wanted, and, as he can feel when the surgeon cannot, he soon understands how to manage the bougie himself, and can take it out, wash it, change it, or replace it, as he pleases. If he is a very restless, fretful, or naturally irritable man, it may prevent sleep, or prove inconvenient, in either of which cases it may be removed for two or three hours, at the pleasure of the individual, whose private affairs may otherwise render this indulgence necessary. It does no harm; it is merely a little delay, which prolongs the time requisite to effect a cure.

"If the pressure is made by a stiff, unyielding instrument, inflammation and ulceration may be the consequence, and many evils may be the result; but then this is the abuse of the practice, not the adoption of it, and forms no part of that which I have recommended. The pressure, according to my views, must be so nicely regulated as to cause absorption, but not to give rise to ulceration; and I firmly believe that it may be graduated in such a manner as to fulfil these intentions most accurately. When the objects stated are duly accomplished, the patient soon perceives that the stream of urine is enlarged, that it comes more freely, and that the general irritation of the bladder and of the neighbouring parts has diminished. The principal and most satisfactory sign of amendment is the more ready flow of the urine; and, although the bougie should not advance, the amendment on this point is often progressive, until at last the bougie is either found to have passed through the stricture, unknown to the patient, or is gently pressed through by his own hand, or by that of the surgeon. The time necessary for the accomplishment of this object must be longer or shorter, according to the extent and nature of the disease, and the state of the constitution of the patient. The object is effected in some cases in from three to six days, in others the progress is slow, although evident, and it may require as many weeks; but in no case that I have met with has the practice failed. When the canal is thus rendered pervious, the cure is only half completed, although the most difficult and dangerous part has been accomplished. The stricture has yielded in its centre, but not in its circumference, and two courses may be pursued: one is, to increase the size of the bougie, so that it may press on the circumference of the anterior part of the stricture, until it causes its removal; the other is effected by passing the bougie through the stricture, and gradually enlarging it: thus pressing on the inner circumference in preference to its anterior surface, which method I prefer.

"When the bougie has passed through the stricture, I always carry it into the bladder, and then replace it by a catheter, the use of which may or may not be continued, at the pleasure of the surgeon. The catheter is not necessary to draw off the urine, as it flows as readily by the side of the bougie; but it proves that the instrument is in the bladder, and it is always a great satisfaction, both to the patient and surgeon, to see the urine flow through it. I have a gentleman now under my care, whose stricture has been overcome in this way, but in whom the point of a small bougie almost always enters into one of the openings of the ejaculatory ducts, and that of a larger one catches on it, and will not often proceed without a little management. If the error be committed of allowing the bougie to lodge in one of these openings, inflammation will in all probability be communicated to the testis, and there is always a chance of such an accident occurring when the orifices of these ducts are irritated, even by the instrument resting upon them. If a catheter is used, the eye of the instrument should pass fairly into the bladder, for half an inch at least, but it is better to use one made with a hole at the extremity, and, when the instrument is not in the bladder, (and it cannot always be borne there,) it should be withdrawn, so as to lie in the membranous part of the urethra, with the point near to, but not irritating the prostate gland. It will often remain in that situation quietly, when it cannot be advanced without producing the greatest irritation. In some instances where this has taken place, the instrument must be altogether withdrawn until it has been subdued, when it may be cautiously replaced. The greatest evil, however, usually arises from increasing the size of the bougie too rapidly: and this is a point to which the greatest attention must be paid, whilst it is the error into which both surgeon and patient most frequently fall, as I have stated, when treating of the cure by dilatation. It is, then, the point which requires the greatest nicety of management; and this can only be acquired by observation founded on experience. The irritability of the inside of the stricture, and the part adjacent but posterior to it, is sometimes greatly augmented by the urine which passes over them. This is frequently diminished in quantity as well as altered in quality, being loaded with salts and other matters, which render it extremely irritating; and, unless it be brought more to its natural state, little or no progress will be made in completing the cure.

"The diet of the patient must then be an object of particular attention, and the urine should be tested from time to time, in order to ascertain its nature; and the general treatment must be strictly continued until it is found to have lost its irritating qualities, when there will be a fair prospect of completing the cure.

"The presence of the bougie gives rise to a discharge, which is greater or less, according to the state of irritability of the patient; but it is never accompanied by pain, unless inflammation is brought on, either by accidental circumstances, or from the size of the bougie being too rapidly increased, so as to distend the canal beyond what is can readily bear." (P. 190.)

Then follow several cases in which this plan of treatment was attended with success, after others had been tried in vain. We consider this to be one of the greatest improvements of the surgery of the urethra of modern times. Henceforward there will be no excuse for using violence in cases of impassable stricture, for we believe that there are few, if any cases, in which a cure may not be effected by long-continued pressure.

In the thirteenth lecture will be found a very good description of the acute diseases of the prostate and prostatic portion of the urethra; but we must pass over these, in order to have sufficient space for the following passages, with which we must conclude this long review:

"The enlargement is sometimes but trifling, in which case the prostate retains its natural shape, and merely projects a little into and around the orifice of the bladder; but, when it is considerable, as in very prolonged and neglected cases, it is often as large as a full-sized orange. One lateral half is usually much larger than the other, and protrudes into the bladder, giving rise to one or more projections, which cause great distress to the individual. The left side, I am led to believe, undergoes this change more frequently than the right, although no satisfactory reason can be given why it should be so; and, whilst one projection is directly backwards and inwards, it sometimes is seen to form a second immediately behind the orifice of the bladder, and which is frequently supposed to be an enlargement of that part of the gland behind the entrance of the vasa deferentia, and which has been called by Sir E. Home the third lobe. Without denying that a third or middle lobe exists, and is occasionally diseased and enlarged, constituting a projection of an apparently similar nature, I am of opinion that it is of more infrequent occurrence than has been supposed. That it is, in fact, not always the third lobe alone in a diseased state, but a continuation of the enlargement of the lateral lobe. In the lithograph drawing, p. 230, a disease of this kind is represented: the third lobe appears to be projecting in a very distinct manner into the bladder immediately behind the orifice of the urethra, and the enlarged left lateral lobe is also seen protruding into it, and forming a second projection by the side of the first. Dissection from behind, however, shows that these two projections are formed by the same part, viz. the left lateral lobe, and that the smaller pyriform one is not formed by a distinct third lobe." (P. 226.)

"The local treatment of the bar or stricture of the neck of the bladder is of the greatest importance; and, as the disease has been only of late distinguished, is not thoroughly understood. Experience has not yet enabled us to support our practice by numerous instances of successful management, although the cases which have fallen under my own observation lead, I trust, to just and legitimate conclusions.

"When the disease occurs in persons under or about the middle period of life, the steady use of the solid silver sound, gradually increasing the size to the largest the urethra will admit, will gradually effect a cure; although, to prevent a relapse, it should be passed occasionally. I have seen, within these few days, one of the first cases to which my attention was drawn, now several years ago. The gentleman is himself a medical man, and was treated by me in this manner, after believing his life to have been in danger, from complete retention of urine for several hours. He assures me he is now free from inconvenience, but passes his solid bougie from time to time, lest he should suffer a relapse.

"I have had, within the last twelvemonth, another medical gentleman, of seventy years of age, under my care, in whom this complaint was commencing, and which he believed to depend on disease of the prostate gland, the symptoms being in many respects similar. I could not, however, on examination, detect any enlargement of the prostate, although some might exist, whilst the contraction of the orifice of the bladder was clearly to be felt. The frequency of making water was great, particularly at night, and the general irritation considerable; a small quantity of urine remaining unevacuated, however great the effort made for its discharge. This gentleman has been restored to health and comfort, and his symptoms all relieved, by the use of the large silver catheter, and by washing out the bladder with water as hot as he could comfortably bear it, every other day. It had a marked and rapid effect upon him; and I am so satisfied of the efficacy of the remedy, and of the advantage to be derived from its use, that I strongly recommend it in all cases in which there is any affection of the bladder; sometimes combined with the various preparations of opium, and particularly those of morphia. I believe it is important that the water should be of a higher temperature than that of the urine when discharged. It should, however, be first used at 98°; and the temperature may be gradually increased until it has reached the highest points that feel comfortable and advantageous to the patient, at which it should be retained. I always inject as much as feels agreeable to him, on similar principles: at first the quantity may be small, but after a time it should be gradually increased; and whilst the bladder loses its irritability, it acquires the capability of being dilated in many instances, in which, from long continued irritation, it had become thickened and contracted. The lotura vesicæ is, in fine, a means of cure which should never be neglected in diseases of the bladder, and which should only be abandoned when it is found on trial to be of no advantage." (P. 271.)

It is unnecessary to add any formal recommendation of lectures which will be read in every country where the English language is known, and which, in spite of some errors, will add even to the distinguished reputation of their author.

